# Exploring the Interplay Between Psychosis and Sleep Disruption: Insights into Course, Insomnia, Nightmares, and Treatment

**DOI:** 10.1192/j.eurpsy.2024.1605

**Published:** 2024-08-27

**Authors:** J. Camilo

**Affiliations:** Psychiatry, CHTMAD, Vila Real, Portugal

## Abstract

**Introduction:**

Psychosis and sleep disruption are complex phenomena that often intertwine, influencing each other in intricate ways. This abstract delves into the dynamic relationship between psychosis and sleep disturbances, shedding light on their course, the prevalence of insomnia, the role of nightmares and dreams, and the impact of psychotic symptoms on sleep patterns. Additionally, it discusses the treatment approaches for individuals with psychosis and sleep disturbances, as well as the consequences of these interventions on both conditions.

**Objectives:**

To investigate the longitudinal course of psychosis and sleep disruption, exploring their temporal connections; to assess the prevalence and characteristics of insomnia among individuals experiencing psychosis; to examine the relationship between nightmares, dreams, and psychotic experiences; to analyze the impact of psychotic symptoms on the pattern and architecture of sleep; to review current treatment modalities for individuals with co-occurring psychosis and sleep disturbances and their effects on both conditions.

**Methods:**

Systematic review

**Results:**

Preliminary findings indicate a bidirectional relationship between psychosis and sleep disruption, with each exacerbating the other over time. Insomnia is prevalent among individuals with psychosis, contributing to the severity of psychotic symptoms. Nightmares and disturbing dreams are common experiences, often mirroring the content of psychotic hallucinations and delusions. Psychotic symptoms disrupt sleep patterns, leading to decreased sleep efficiency and altered sleep architecture. Various treatment approaches show promise in addressing both psychosis and sleep disturbances, but further research is needed to determine their long-term effects.

**Conclusions:**

The intricate interplay between psychosis and sleep disruption, emphasizing the need for a holistic approach to assessment and intervention. Understanding the course of these conditions, the high prevalence of insomnia, and the role of nightmares and dreams in the psychotic experience is crucial for developing targeted interventions. Additionally, recognizing the impact of psychotic symptoms on sleep patterns is vital for improving overall well-being. Effective treatment strategies that address both psychosis and sleep disturbances offer hope for enhanced outcomes, but ongoing research is essential to fully elucidate their potential benefits and long-term consequences.

**Disclosure of Interest:**

None Declared

